# Multiple physical symptoms in a military population: a cross-sectional study

**DOI:** 10.1186/1744-859X-12-24

**Published:** 2013-07-17

**Authors:** Varuni A de Silva, Nicholas ELW Jayasekera, Raveen Hanwella

**Affiliations:** 1 Department of Psychological Medicine, Faculty of Medicine, University of Colombo, Colombo 08, Sri Lanka; 2 Directorate of Health Services, Sri Lanka Navy, Colombo, Sri Lanka

**Keywords:** Trauma, Stress, Stress disorders, Post-traumatic, Military personnel, Special forces, War, Sri Lanka

## Abstract

**Background:**

Medically unexplained symptoms have been reported among both civilians and military personnel exposed to combat. A large number of military personnel deployed to the Gulf War in 1991 reported non-specific symptoms. These symptoms did not constitute a clearly defined syndrome. Post-traumatic stress disorder (PTSD) and to a lesser degree exposure to combat are associated with physical symptoms.

**Methods:**

This is a cross-sectional study of representative samples of Sri Lanka Navy Special Forces and regular forces deployed in combat areas continuously during a 1-year period. Multiple physical symptoms were elicited using a checklist of 53 symptoms. Cases were defined as individuals with ten or more symptoms. Symptoms of common mental disorder were identified using the General Health Questionnaire 12 (GHQ-12). PTSD was diagnosed using the 17-item National Centre for PTSD checklist civilian version.

**Results:**

Prevalence of multiple physical symptoms was 10.4% (95% CI 8.11–12.75). Prevalence was significantly less in the Special Forces (5.79%) than in the regular forces (13.35%). The mean number of symptoms reported by those who met the criteria for PTSD was 12.19 (SD 10.58), GHQ caseness 7.87 (SD 7.57) and those without these conditions 2.84 (SD 3.63). After adjusting for socio-demographic and service variables, ‘thought I might be killed’ , ‘coming under small arms fire’ , and ‘coming under mortar, missile and artillery fire’ remained significant. Multiple physical symptoms were associated with functional impairment and poor perceived general health.

**Conclusions:**

Prevalence of multiple physical symptoms was significantly lower in the Special Forces despite high exposure to potentially traumatic events. More multiple physical symptoms were reported by personnel with PTSD and common mental disorders. Multiple physical symptoms were associated with functional impairment.

## Background

Medically unexplained symptoms (MUS) have been reported among both civilians and military personnel exposed to combat. These symptoms can be classified into different syndromes such as somatoform disorder, chronic fatigue syndrome, and conversion disorder. There is considerable overlap between these syndromes [1]. Symptoms such as abdominal distension, headache, and weakness are present in many such syndromes. The term, medically unexplained symptoms, is used when there is no objective evidence of an illness despite subjective complaints of symptoms. Only a small proportion, about one in four, seeks medical attention for their symptoms [2]. Factors that precipitate seeking medical help include perceived seriousness, functional impairment, and psychological distress [1].

There is overlap between multiple physical symptoms and psychological morbidity [1,3]. Physical symptoms are associated particularly with anxiety and mood disorders. The likelihood of a psychiatric disorder increases with increasing numbers of physical symptoms [4]. In people with MUS, psychological morbidity increases risk of medical consultations and functional impairment [1,4].

A large number of military personnel deployed to the Gulf War in 1991 reported non-specific symptoms [5,6]. These symptoms did not constitute a clearly defined syndrome. Initially, these symptoms were attributed to physical conditions such as exposure to chemicals or vaccination. However, there is little evidence to support this [7,8]. Department of Defense Comprehensive Clinical Evaluation Program, which evaluated 18,495 Gulf War veterans, found that joint pain, fatigue, headache, memory problems, and sleep disturbance were the most frequently reported symptoms [7]. Studies of non-combatants such as victims of sexual violence, civilians in war situations, and fire fighters also show an increased incidence of multiple physical symptoms [9-11].

Findings from Gulf War veterans suggest that psychological factors may be implicated in the etiology of the unexplained physical symptoms [12]. Exposure to trauma is associated with post-traumatic stress disorder (PTSD), and those with PTSD are more likely to report physical symptoms. Those who do not meet the criteria for PTSD but experience post-traumatic stress symptomatology also report increased physical symptoms [13]. In people exposed to war situations, depression is also associated with unexplained physical symptoms [14,15].

Most of the evidence regarding the association of combat exposure and unexplained physical symptoms are from studies of military personnel in the USA and the UK. Studies from military personnel from different ethnic and cultural backgrounds are important to determine if these findings are applicable in different settings. Therefore, we carried out a study of Sri Lanka Navy (SLN) personnel, which included both regular forces and Special Forces to assess the mental health outcomes in those deployed in combat areas.

The Sri Lanka Defense Forces have been engaged in combat operations for the last 30 years. In 2006, the level of combat operations intensified as reflected in the casualty figures. During the period 2006–2009, 190 officers and 5,700 other ranks of the Sri Lanka Army were killed and 27,000 injured [16]. During this period, 485 personnel from the SLN were killed and 245 permanently disabled [17]. The conflict ended in 2009. The overall exposure to potentially traumatic events was high [18]. Exposure to potentially traumatic events was significantly greater in the Special Forces. More than 60% in both groups had seen dead or injured persons. More than 80% of the Special Forces reported discharge of weapons in direct combat compared to 26.7% of the regular forces. Among the Special Forces, 81.5% had engaged in combat with enemy vessels compared to 29.4% of the regular forces.

## Methods

### Study sample

A cross-sectional study was carried out among SLN Special Forces and regular forces deployed in combat areas. The data collection commenced 3 months after the end of combat operations. The study methods are described in detail in a previous publication [18]. Representative samples of Special Forces and regular forces were selected using simple random sampling. The sampling frames used were the lists of personnel from the navy central database. Samples were selected using computer-generated random numbers. Only personnel who had served continuously in combat areas during the 1-year period prior to the end of combat operations were included in the study. Since there were no females in the Special Forces, females were excluded from the regular forces group. A total of 259 Special Forces and 412 regular navy personnel were recruited to the study. The response rate was 93.8%. The rate of missing values for individual items in the survey was about 10%. These questionnaires were excluded from analyses of the relevant variables.

### Outcome measures

The questionnaire used in the study ‘Health of UK military personnel deployed to the 2003 Iraq war’ was used as the data collection instrument [19]. Permission was obtained from the authors for the use of the questionnaire. [20]. Multiple physical symptoms were elicited using a checklist of 53 symptoms, which have been used in previous studies of military personnel [19,21]. Cases were defined as individuals with ten or more symptoms. This case definition represents the top decile of this sample.

### Measures of mental health

Symptoms of common mental disorder were identified using the General Health Questionnaire 12 (GHQ-12), and cases were defined as individuals scoring 4 or more. PTSD was diagnosed using the 17-item National Centre for PTSD checklist civilian version, and cases were defined as individuals scoring 50 or more [22].

### Exposure to potentially traumatic events

Exposure to potentially traumatic events was assessed using ten questions. These identified discharging weapons in direct combat, thinking might be killed, seeing dead or wounded, handling dead bodies, aiding wounded, coming under small arms, mortar, missile or artillery fire, experiencing landmine strikes, and experiencing hostility from civilians and combat with enemy vessels.

### Functional impairment

Functional impairment was assessed with five questions from the SF-36 [23]. These questions explored the perceived functional impairment related to physical health or emotional problems. The areas explored were interference with normal social activities, problems with work or other regular daily activities as a result of physical health, cutting down on the amount of time spent on work or other activities, accomplishing less than would like, limited in the kind of work or difficulty performing work or other activities.

### Ethical approval

Ethical clearance was obtained from the Ethics Review Committee of the Faculty of Medicine, University of Colombo. Participation was voluntary and written informed consent was obtained from all participants. The questionnaire did not identify the participants by name.

### Statistical analyses

Analyses were done using SPSS version 13.0. We looked for association between multiple physical symptom cases and demographic variables, indicators of psychological morbidity, and functional impairment. Pearson's χ^2^ test was used to assess the difference in demographic characteristics between Special Forces and regular forces. Correlation between the mean number of symptoms and scale scores was assessed with the Spearman correlation coefficient. Logistic regression analysis was used to calculate unadjusted and adjusted odds ratios (OR) with 95% confidence limits (95% CI). Model adequacy was tested using goodness of fit with the Hosmer-Lemeshow test. In assessing the association between multiple physical symptoms and exposure to potentially traumatic events, we adjusted for the socio-demographic variables age, education, marital status, rank, service type, and role within the unit. In the analysis for the association between multiple physical symptoms and functional status, the first model adjusted for age, education, marital status, rank, service type, and role within the unit and the second model adjusted for these variables and PTSD.

## Results

The socio-demographic characteristics of the sample have been described in a previous publication [18]. The sample consisted of 259 Special Forces and 412 regular navy personnel (Table 1). The mean age of the sample was 27.6 years (SD 5.02). Three hundred twenty nine (49.0%) were single, 333 (49.6%) were married, and 2 (0.3%) were previously married. There were 33 (4.9%) commissioned officers, 104 (15.5%) non-commissioned officers, and 534 (79.6%) other ranks. Two hundred thirty six (35.2%) were engaged in combat duty, 195 (29.1%) served on board naval vessels, and 237 (35.3%) were engaged in non-combat duties, which included medical, logistic, engineering, communication, and administrative roles.

**Table 1:** Description of study sample

	**Special forces**	**Regulars**	**Significance**
	**n = 259 (%)**	**n = 412 (%)**	
Age (years)			
0–24	102 (39.4)	96 (23.3)	χ^2^ = 24.8, df = 4, p < 0.001
25–34	146 (56.4)	277 (67.2)	
>35	11 (4.2)	39 (9.5)	
Marital status			
Married/divorced	101 (39.6)	234 (57.2)	χ^2^ = 19.8, df = 2, p < 0.001
Never married	154 (60.4)	175 (42.8)	
Educational status			
Less than GCE O-level	137 (53.5)	104 (25.4)	χ^2^ = 59.2, df = 3, p < 0.001
GCE O-level	91 (35.5)	195 (47.7)	
GCE A-level or higher	28 (10.9)	110 (26.9)	
Rank (current)			
Commissioned officer	14 (5.4)	19 (4.6)	χ^2^ = 38.4, df = 2, p < 0.001
Non-commissioned officer	68 (26.3)	36 (8.7)	
Non-combat duties	177 (68.3)	357 (86.7)	
Role within unit			
Land combat	183 (70.7)	53 (13.0)	χ^2^ = 257.0, df = 2, p < 0.001
On board naval vessels	60 (23.2)	135 (33.0)	
Non-combat duties	16 (6.2)	221 (54.0)	

Prevalence of multiple physical symptoms was 10.4% (95% CI 8.11–12.75). Prevalence was less in the Special Forces (5.79%) than in the regular forces (13.35%). Association between multiple physical symptoms and demographic variables are shown in Table 2. Unadjusted odds ratios found that those Special Forces personnel had significantly lower odds of developing multiple physical symptoms compared with regular forces (OR 0.40 (95% CI 0.22–0.72)). This significant association disappeared when we adjusted for demographic variables in model 1 and demographic and psychological morbidity in model 2. Unadjusted odds ratios showed that those with education level of General Certificate of Education (GCE) O-level or above had significantly higher odds of multiple physical symptoms. Personnel engaged in non-combat duty had significantly higher odds of developing multiple physical symptoms than those engaged in combat duty. The significant association with non-combat duty and educational status disappeared in model 1 (adjusted for demographic variables) and model 2 adjusted for demographic and psychological morbidity.

**Table 2:** Association between multiple physical symptoms and socio-demographic variables

	**Mean number of symptoms (SD)**	**Unadjusted OR (95% CI)**	**Model 1 adjusted OR (95% CI)**	**Model 2 adjusted OR (95% CI)**
Service type				
Special Forces	2.92 (3.98)	0.40 (0.22–0.72)	0.54 (0.25–1.18)	0.66 (0.29–1.49)
Regular forces	4.12 (5.21)	1.0	1.0	1.0
Age (years)				
0–24	3.58 (4.26)	1.0	1.0	
25–34	3.55 (4.81)	1.39 (0.77–2.52)	0.99 (0.50–1.98)	1.24 (0.57–2.68)
>35	4.84 (6.50)	2.17 (0.87–5.40)	1.12 (0.34–3.65)	1.55 (0.43–5.59)
Marital status				
Never married	3.32 (4.33)	1.0	1.0	1.0
Married/divorced	3.94 (5.13)	1.65 (0.99–2.74)	1.43 (0.77–2.65)	1.70 (0.86–3.37)
Educational status				
Less than GCE O-level	3.13 (4.32)	1.0	1.0	
GCE O-level	3.64 (4.64)	1.90 (1.02–3.53)	1.66 (0.86–3.21)	1.48 (0.73–2.99)
GCE A-level or higher	4.50 (5.70)	2.41 (1.21–4.83)	1.60 (0.70–3.65)	1.63 (0.67–3.94)
Rank (current)				
Commissioned officer	4.91 (4.35)	1.56 (0.58–4.19)	1.42 (0.56–5.19)	1.42 (0.44–4.63)
Non-commissioned officer	3.47 (5.10)	0.93 (0.46–1.89)	0.82 (0.33–2.01)	0.97 (0.37–2.55)
Other ranks	3.58 (4.75)	1.0	1.0	1.0
Role within unit				
Land combat	3.23 (3.76)	1.0	1.0	
On board naval vessels	3.14 (4.91)	1.23 (0.61–2.48)	0.82 (0.33–2.01)	0.88 (0.39–2.01)
Non-combat duties	4.43 (5.46)	2.16 (1.17–4.0)	1.13 (0.51–2.51)	0.95 (0.42–2.16)
Mental health outcomes				
GHQ case (yes)	9.57 (7.57)	9.93 (5.69–17.32)	9.68 (5.31–17.64)	8.43 (4.54–15.66)
PTSD case (yes)	12.19 (10.58)	9.57 (3.47–26.37)	9.48 (3.13–28.76)	3.54 (1.03–12.17)

Model 1—adjusted for age, education, marital status, rank, role within unit, and service type. Model 2—adjusted for age, education, marital status, rank, service type, role within unit, GHQ, and PTSD.

The mean number of symptoms reported by those who met the criteria for PTSD was 12.19 (SD 10.58), GHQ caseness 7.87 (SD 7.57), and those without these conditions 2.84 (SD 3.63). There was significant correlation between the number of physical symptoms and PTSD scale scores (r = 0.671) and GHQ scores (r = 0.469). Multiple physical symptom caseness was associated with PTSD (adjusted OR 3.54 (95% CI 31.03–12.17)) and GHQ caseness (adjusted OR 8.43 (95% CI 4.54–15.66)).

Exposure to potentially traumatic events was assessed using ten questions (Table 3). Unadjusted odds ratios showed that two types of traumatic exposure ‘thought I might be killed’ (OR 2.18 (95% CI 1.32–3.61)) and ‘coming under mortar, missile and artillery fire’ (OR 1.73 (95% CI 1.05–2.85)) were significantly associated with multiple physical symptoms. After adjusting for age, education, marital status, rank, service type, and role within the unit, ‘thought I might be killed’ , ‘coming under small arms fire’ , and ‘coming under mortar, missile and artillery fire’ were significant. These events can be classified as ‘risk to self’ events [24,25].

**Table 3:** Association between multiple physical symptoms and exposure to potentially traumatic events

**Combat exposure**	**Mean number of symptoms (SD)**	**Unadjusted OR (95% CI)**	**Adjusted OR (95% CI)**^**a**^
Discharged weapon in direct combat	3.31 (4.60)	1.57 (0.95–2.59)	1.09 (0.58–2.08)
Thought I might be killed	4.38 (5.20)	2.18 (1.32–3.61)	2.67 (1.56–4.58)
Seeing dead or wounded	3.70 (5.10)	1.12 (0.65–1.94)	1.25 (0.70–2.28)
Handled bodies	3.81 (5.12)	1.22 (0.74–2.01)	1.33 (0.76–2.25)
Aided wounded	3.94 (4.93)	1.0 (0.60–1.67)	1.12 (0.64–1.96)
Came under small arm fire	3.81 (5.05)	1.32 (0.8–2.17)	1.97 (1.10–3.53)
Came under mortar, missile, artillery fire	4.29 (5.95)	1.73 (1.05–2.85)	2.21 (1.27–3.89)
Experienced landmine strikes	3.92 (5.99)	0.74 (0.17–3.20)	0.82 (0.18–3.63)
Experienced hostility from civilians	5.44 (7.0)	2.54 (0.81–7.95)	3.05 (0.93–10.0)
Involved in combat with enemy vessels	3.45 (4.86)	0.74 (0.45–1.22)	1.12 (0.61–2.06)
Health limiting participation in vigorous activities	6.42 (6.72)	5.12 (3.04–8.63)	5.01 (2.52–9.97)

^a^Adjusted for age, education, marital status, rank, service type, and role within the unit.

Association between multiple physical symptoms and functional impairment is shown in Table 4. Multiple physical symptoms were initially associated with all indicators of impaired functioning. When we adjusted for demographic and service variables, ‘health interfered with social life’ , ‘accomplished less than would like,’ ‘limited in type of work’ , ‘difficulty performing work’ , and ‘participation in vigorous activities’ remained significant. After adjusting for demographic variables and PTSD ‘health interfered with social life’ (adjusted OR 2.71 (95% CI 1.57–4.66)), ‘accomplished less than would like’ (adjusted OR 1.96 (95% CI 1.03–3.72)), ‘limited in type of work’ (adjusted OR 2.35 (95% CI 1.27–4.33), ‘difficulty performing work’ (adjusted OR 3.34 (95% CI 1.94–5.77)), and ‘participation in vigorous activities’ (adjusted OR 4.63 (95% CI 2.63–8.16)) remained significant.

**Table 4:** Association between multiple physical symptoms and functional impairment

	**OR (95% CI)**	**Model 1 adjusted OR (95% CI)**	**Model 2 adjusted OR (95% CI)**
Health interfered with social life	3.21 (1.93–5.34)	2.96 (1.74–5.04)	2.71 (1.57–4.66)
Cut down time on work/other activities	2.17 (1.16–4.06)	1.87 (0.97–3.60)	1.67 (0.85–3.29)
Accomplished less than would like	2.14 (1.17–3.94)	2.11 (1.13–3.95)	1.96 (1.03–3.72)
Limited in type of work	2.53 (1.43–4.47)	2.47 (1.35–4.49)	2.35 (1.27–4.33)
Difficulty performing work	3.87 (2.33–6.44)	3.76 (2.21–6.38)	3.34 (1.94–5.77)
Health limiting participation in vigorous activities such as running	5.12 (3.04–8.63)	4.87 (2.79–8.50)	4.63 (2.63–8.16)
Rating of general health as poor or very poor	4.42 (2.53–7.74)	4.34 (2.42–7.79)	4.05 (2.22–7.37)

Model 1—adjusted for age, education, marital status, rank, service type, and role within the unit. Model 2—adjusted for age, education, rank, marital status, service type, role within the unit, and PTSD.

There was significant difference between personnel with and without multiple physical symptoms in their rating of general health as poor or very poor. This remained significant after adjusting for demographic and service variables and PTSD (adjusted OR 4.05 (95% CI 2.22–7.37)). Personnel with multiple physical symptoms were more likely to report that they seem to get ill more frequently than others (adjusted OR 5.79 (95% CI 2.97–11.28)), and they expect their health to get worse (adjusted OR 4.24 (95% CI 2.36–7.65)).

## Discussion

Of the Sri Lanka Navy personnel deployed in combat areas, 10.4% reported ten or more physical symptoms. The prevalence was lower in the Special Forces than in the regular forces. There was significant association between multiple physical symptoms and PTSD and psychological morbidity. Multiple physical symptoms were associated with functional impairment.

Although unadjusted odds ratios showed significantly higher odds for those with higher educational level and non-combat personnel, these disappeared when we adjusted for service type. We found that regular forces were more likely than Special Forces to have higher educational level and be engaged in non-combat duty. Therefore, the association seen in the unadjusted analysis was due to a confounding factor.

In this study, the definition of a case of multiple physical symptoms was based on the top decile of the sample. In the study of UK military personnel, which used the same symptom list, the cases were defined as having 18 or more symptoms [19]. When we used a cutoff of 18 symptoms, prevalence was 1.2% in the Special Forces and 2.9% in the regular forces which is much lower than in the UK sample. A study of 21,244 Gulf War veterans reported that 8.6% experienced ten or more symptoms which is similar to the prevalence in our sample [26]. Prevalence of psychological morbidity such as PTSD, common mental disorders, fatigue, hazardous drinking, and smoking was also low in our sample [18,27,28]. Because PTSD and common mental disorders are probably involved in the etiology of multiple physical symptoms, lower rates of these conditions among SLN personnel could have influenced the rates of multiple physical symptoms.

It is interesting that the Special Forces reported lower rates of multiple physical symptoms compared to regular forces. They also had lower rates of fatigue and common mental disorders [18]. Because Special Forces are exposed to more potentially traumatic events than regular forces, it may be assumed that they have higher rates of mental health problems. The ‘healthy warrior’ effect has been previously suggested as an explanation for the low rates of mental health problems among deployed military personnel [29]. It suggests that psychologically unfit personnel drop out during training, and therefore, those deployed are healthier. This can explain the lower rates of multiple physical symptoms in the Special Forces. With the associated functional impairment, it is unlikely that personnel with high rates of physical symptoms can function satisfactorily in the Special Forces. In the regular forces too, those performing combat roles had less physical symptoms. Special Forces personnel such as Royal Marine Commandos and paratroopers were noted to have lower rates of multiple physical symptoms, fatigue, and general mental health problems [30]. Apart from the ‘healthy warrior’ effect, rigorous selection and training of Special Forces personnel may also account for their increased resilience.

Comparison of prevalence of multiple physical symptoms is difficult because of the difference in the diagnostic criteria. Therefore, it may be more meaningful to compare the mean number of physical symptoms across studies. Gulf War veterans diagnosed with PTSD reported an average of 6.7 (SD 3.9) physical symptoms, those with a non-PTSD psychological condition 5.3 (SD 3.5), those with medical illness 4.3 (SD 3.4), and a group diagnosed as ‘healthy’ 1.2 (SD 2.2) [26]. These are similar to our findings where the mean number of symptoms reported in personnel with PTSD was 12.19 (SD 10.58), common mental disorders 7.87 (SD 7.57), and those without these conditions 2.84 (SD 3.63). Physical symptoms are also known to be associated with post-traumatic stress symptomatology [13]. Our study supports this observation because there was significant correlation between the number of physical symptoms and PTSD scale score (r = 0.671). The correlation between physical symptoms and PTSD symptoms is an important finding because many personnel did not meet the criteria for PTSD although they reported symptoms of PTSD.

Exposure to traumatic stress itself has been associated with unexplained physical symptoms and, to a lesser degree, poor physical health although [31]. In our study ‘thought I might be killed,’ ‘coming under small arms fire,’ and ‘coming under mortar, missile and artillery fire’ were significantly associated with multiple physical symptoms, even after adjustment for socio-demographic variables. These events can be classified as ‘risk to self’ events [24,25]. ‘Risk to life events’ are more strongly associated with PTSD than risk to other events such as witnessing dead or injured [24].

Multiple physical symptoms are associated with functional impairment, and this association remained after adjusting for demographic factors and PTSD. This is important because reports of functional impairment and the perceived ill health in personnel with physical symptoms indicate that they are not functioning optimally.

Several mechanisms could explain the increased physical morbidity in people with PTSD. The biological mechanisms suggested are cardiovascular reactivity, autonomic hyperarousal, disturbed sleep physiology, adrenergic dysregulation, immune dysregulation, enhanced thyroid function, and altered HPA activity [14]. In addition to biological mechanisms, psychological correlates such as depression and poor health habits such as smoking and drinking can also result in increased physical morbidity.

This is a study of military personnel from a different cultural background to those in the USA or the UK. Social factors such as separation from home and family, cohesion and support from members of the unit, and acceptability by the community can modify stressful experience. These factors must be explored in future studies.

The strengths of this study are the large, randomly drawn sample of military personnel with high exposure to potentially traumatic events and the very high response rate. There are several limitations of this study. Self reports were used in the assessment, and this can result in underreporting of symptoms specially because military personnel may be reluctant to acknowledge the presence of symptoms. Personnel with multiple physical symptoms may also overreport symptoms. The study reports the presence of multiple physical symptoms rather than medically unexplained symptoms because no attempt was made to identify the etiology of symptoms. A cross-sectional study of this nature cannot establish causation. Future studies should assess the prevalence of symptoms before and after deployment to identify the symptom burden which can be attributed to deployment.

## Conclusions

This study found that the prevalence of multiple physical symptoms of 10.4% was lower than that reported in other studies of military personnel. The prevalence was lower in the Special Forces than in the regular forces although Special Forces were exposed to more potentially traumatic events. There was significant association between multiple physical symptoms and PTSD and psychological morbidity. Multiple physical symptoms were associated with functional impairment. Although it is known that PTSD and depression are associated with functional impairment in military personnel, this study found that multiple physical symptoms are also associated with significant functional impairment. Therefore, programs screening for adverse mental health outcomes in deployed service personnel should also include screening for multiple physical symptoms.

## Competing interests

The authors declare that they have no competing interests.

## Authors' contributions

VAdS and RH contributed to the design of the project, supervision of data collection, analysis of data, and writing of the paper. NELWJ contributed to the design of the project and supervision of data collection. All authors read and approved the final manuscript.

## Authors' information

VAdS and RH are civilians who provide honorary clinical services to the Sri Lanka Navy. NELWJ is the Director General of Health Services in the Sri Lanka Navy.
